# The experience of giving birth: a prospective cohort in a French perinatal network

**DOI:** 10.1186/s12884-022-04727-7

**Published:** 2022-05-26

**Authors:** Chloé Arthuis, Juliette LeGoff, Marion Olivier, Anne-Sophie Coutin, Nathalie Banaskiewicz, Philippe Gillard, Guillaume Legendre, Norbert Winer

**Affiliations:** 1 Service de Gynécologie Obstétrique, UMR 1280, Centre Hospitalier Universitaire de Nantes, CIC Et Hôpital Mère-Enfant-Adolescent, NUN, INRAE, PhAN, 44000 Nantes, France; 2 Université de Nantes, Physiologie des Adaptations Nutritionnelles 38 boulevard Jean Monnet, 44000 Nantes, France; 3 Réseau Sécurité Naissance - Naître Ensemble, Réseau de Santé Périnatale Des Pays de La Loire, 3 rue Marguerite Thibert, 44200 Nantes, France; 4 Service de Gynécologie Obstétrique, Centre Hospitalier Universitaire d’Angers, 4 rue Larrey, 49933 Angers, France

**Keywords:** Childbirth experience, Pregnancy, Obstetric violence, Obstetric consent

## Abstract

**Background:**

To assess women's positive and negative perceptions after giving birth. The secondary objectives were to identify the women who had a negative perception of their delivery, define the risk factors, and propose actions that maternity units can take to improve their management.

**Methods/design:**

This study was a multicenter, prospective cohort, conducted in 23 French maternity units constituting one perinatal network, in 2019. All adult women who understood French and gave birth between February 1 and September 27, 2019, were eligible. The exclusion criterion was the woman's objection to participation. Validated self-administered questionnaire (QACE) was sent by email 6 weeks after the child's birth. The main outcome was the experience of childbirth, assessed on a scale of 0 to 10. A good experience was defined by a score ≥ 8/10, and a poor experience by a score < 5. A multinomial logistic regression model, expressed by cumulative proportional odds ratios, were used to determine the factors that might have affected women's experiences during childbirth.

**Results:**

Two thousand one hundred and thirty-fifth women completed the questionnaire, for a participation rate of 49.6%. Overall, 70.7% (*n* = 1501/2121) of the women reported a good experience, including 38% (*n* = 807/2121) who graded their experience with the maximum score of 10. On the other hand, 7.3% (*n* = 156) of the women reported a poor experience. Vaginal delivery (aOR 3.93, 95%CI, 3.04–5.08) and satisfactory management (aOR 11.35 (7.69–16.75)) were the principal determining factors of a positive experience. Epidural analgesia increased the feeling of failure (aOR 5.64, 95%CI, 2.75–13.66). Receiving information and being asked for and agreeing to consent improved the global experience (*P* = 0.03).

**Conclusion:**

The Identikit picture of the woman associated with a poor experience of childbirth shows a nullipara who had a complication during her pregnancy, gave birth after induction of labor, or by cesarean or operative vaginal delivery, with the newborn transferred for pediatric care, and medical management considered unsatisfactory.

## Introduction

Pregnancy and childbirth constitute an essential stage in a woman's life; they bring with them profound physiological and psychosocial changes [1] that must be considered to be a major stress factor. Health care professionals therefore play a key role in supporting the woman and the couple through this stage. Childbirth requires on the one hand the application of a treatment protocol aimed at improving practices and safeguarding women's health [2, 3]. On the other hand, it creates human interactions and trust between women and those caring for them. Both, of course, are integral parts of medical management [3]. The woman’s experience of childbirth is an important quality measure in obstetric care [4]. In France and throughout the world, women have denounced a negative experience of childbirth sometimes even qualified. From 5 to 20% of women describe their experience of delivery as negative [5, 6]. In a European study published in 2015, one pregnant woman in five reported receiving abuse or poor treatment during perinatal care [7]. A systematic review of the literature in 2018 about obstetric violence collected 24 publications from 2007 through 2017; 75% of the studies came from Latin American countries [8]. It characterized the different types of violence denounced by women. The legal framework provides some degree of autonomy for the patient in medical decisions and requires that physicians provide information and obtain the patient's voluntary and informed consent to perform any medical procedure. Most often, "obstetric violence" appears to be used to describe a failure to listen, a lack of information, deprivation of freedom, restriction of movement, or procedures imposed on women. These factors produce an increasingly negative perception of childbirth that can reach the level of trauma. A negative perception of delivery may be a risk factor for developing maternal fear of childbirth [9], postpartum depression [10] "posttraumatic stress disorders" (PTSD) associated with childbirth ranging from 1.3 to 6% [11, 12]. It is possible that health care providers, claiming scientific and technical justifications, sometimes abandon some parts of the information and consent required, especially in emergency situations, as childbirth can sometimes be.

Nonetheless, the data available to characterize this negative perception of childbirth or acts of violence and estimate the proportion of women concerned are sparse. Assessing our practices is essential not only for understanding this phenomenon of "obstetric violence" and thus improving the relationships between women and the teams caring for them, but also for improving women's understanding of medical procedures.

The objective of our study was to assess the positive and negative perceptions of women giving birth in one of the 23 maternity units in the Pays de la Loire region of France. The secondary objectives were to identify the women with a negative perception of their delivery, to define the risk factors for this perception, and to propose actions that maternity units can take to improve women's experience of care.

## Material and methods

### Population

Adult women who understood French were eligible if they gave birth between February 1 and September 27, 2019, in one of the 23 maternity units—public or private, and regardless of level of care—in the Pays de la Loire region. Recruitment was based on a predefined study period with all maternity units in the network. In France, during the study, there were 471 maternity hospitals and 9 midwife led units. There were no birth centers in our network. Those with an in utero fetal death or a termination of pregnancy could also participate. The exclusion criterion was the woman's objection to participation. This cohort study was performed according to the STROBE statement.

### Questionnaire

This study was prospective and observational. We studied the experience of childbirth on a scale of 0 to 10 with the French-Swiss Questionnaire for Assessing the Childbirth Experience (QACE), which has been validated in French [13]. The questionnaires were sent by email 6 weeks after the women gave birth, that is, from March 15 through November 8, 2019. This multidimensional questionnaire was developed to identify women who had a negative experience while giving birth. We added additional questions about the women's social and demographic characteristics and the course of their pregnancy and delivery to the QACE. The initial questionnaire comprised 26 questions, while that completed by the women in this study had 48. The QACE categorized its questions into four dimensions: emotional state (3 items), relationships with staff (4 items), the first moments with the newborn (3 items), and later feelings, a month or more postpartum (3 items). At the end of the questionnaire, we added an open question for free comments. The questionnaire was considered interpretable when the woman had answered enough questions for 80% of the variables to be available. A good experience was defined by a score ≥ 8/10, as defined in the article validating the questionnaire [13], and a poor experience by a score < 5.

### Statistical analyses

All analyses were performed with R software, version 3.6.2. Significance was set at 5%. We conducted a descriptive analysis of the data. The categorical (qualitative) variables were described as percentages, and the continuous (quantitative) variables by their means and standard deviations. For comparative analyses of the categorical variables, we used the χ2 method or Fisher's exact test, as appropriate. A factorial analysis was performed to assess a possible modification effect or interaction between variables. A bivariate and then multivariable analysis with an ordered multinomial logistic regression model, expressed by cumulative proportional odds ratios (ORs), were used to determine the factors that might have affected women's experiences during childbirth. Because the dependent variable to be explained, the "experience of childbirth", was an ordered categorical variable, it was recoded in three ordered classes to define a categories of experience: good (8 to 10), medium (5 to 7), and poor (0 to 4). To identify the associations between the variables and study the variability between women (their similarities and differences), we performed a multiple correspondence analysis.

## Results

**Fig. 1:**
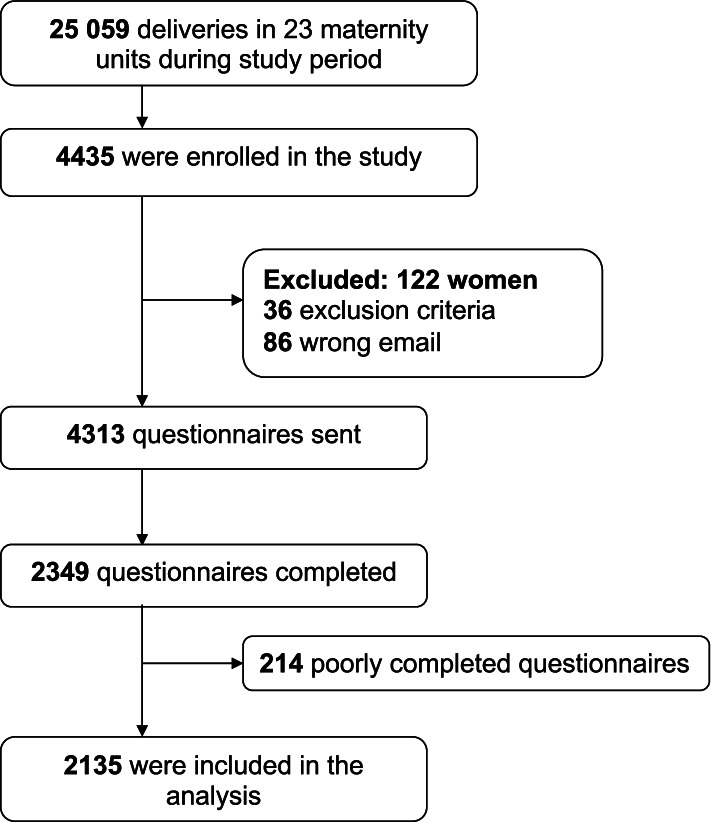
Flow chart of the study population

We received 2135 questionnaires, for a participation rate of 49.6% (Fig. 1), at a mean of 8 weeks after the women had given birth. This figure represents 8.5% of the women who gave birth in network facilities during the study period. The proportion of missing data for each variable in the questionnaire was less than 2%. Accordingly, no imputation of missing data w Indeed, vaginal deliveries and CSection are two different populations regarding the practices carried out, which have a different impact on the experience. as performed. Table 1 summarizes the population's characteristics. The C-section rate was 14.4%, which was comparable to the general population rate in France of 18.9%. Most women gave birth in a level 2 maternity ward (59.6%) and in a public establishment (72.2%).

**Table 1:** Population characteristics

Variables	Women N (%)
**Age (years):**	30.8 [19–45]
**Socio-occupational category (%):**	
- Office, sales, and service staff	1031 (48.4%)
- Manager	366 (17.2%)
- Intermediate professional	363 (17.1%)
- No occupation	200 (9.4%)
- Tradesperson, shopkeeper, small-business owner	92 (4.3%)
- Blue-collar	61 (2.9%)
- Farmer	15 (0.7%)
**Maternity unit status (%):**	
- Private	524 (24.7%)
- Public	1534 (72.2%)
- Private nonprofit	67 (3.2%)
**Maternity unit level (%):**	
- 1	512 (24.1%)
- 2	1267 (59.6%)
- 3	346 (16.3%)
**Number of deliveries/year (%):**	
- < 1000	440 (20.7%)
- 1000 to 3000	1089 (51.2%)
- > 3000	596 (28.1%)
Parity (%):	
- Nulliparous	943 (44.2%)
- Parous	1192 (55.8%)
**Pregnancy (%):**	
- Singleton	2081 (98.6%)
- Twin	28 (1.3%)
- Triplet or more	1 (0.1%)
**Time until first consultation at the maternity ward (%):**	
- < 6th month	1053 (49.4%)
- > or = 6th month	1005 (47.2%)
- No contact	55 (2.6%)
- Don't remember	17 (0.8%)
**Characteristics of the delivery (%):**	
**Day/Night**^a^	983 (46.1%) / 1148 (53.9%)
**Epidural analgesia**	1696 (80.0%)
**Vaginal**	1826 (85.6%)
**Cesarean:**	307 (14.4%)
- Scheduled	78 (25.4%)
- During labor	229 (74.5%)^b^
**Instrumental vaginal delivery:**	311 (17.1%)
- Vacuum extraction	216 (69.5%)
- Forceps or Spatulas	129 (41.5%)
**Episiotomy**	247 (13.5%)
**Induction of labor:**	523 (24.6%)
- Cervical ripening	296 (13.9%)
- Induction by oxytocin	227 (10.7%)
**Transfer of the newborn (%)**	54 (2.6%)
**Birth plan (%):**	
- Verbal	587 (27.5%)
- Written	197 (9.2%)

^a^Daytime: between 8 and 18 h. Night: between 18 and 20 h; XX

^b^Including 25 (8.1%) that were scheduled but took place at a different time

**Fig. 2:**
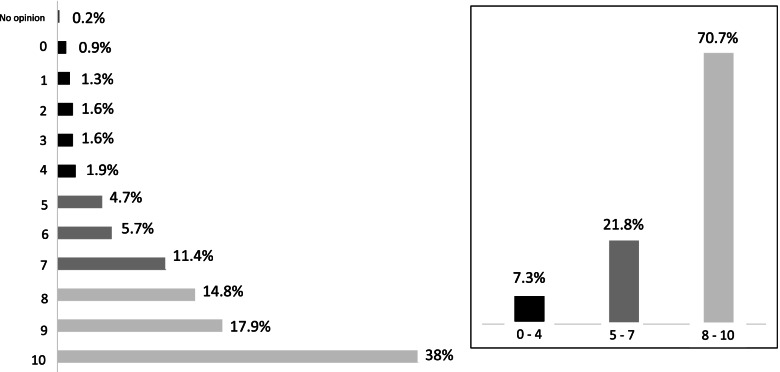
The experience of childbirth analysis on a scale of 0 to 10 with the validate French-Swiss Questionnaire for Assessing the Childbirth Experience (QACE). On the left side, the diagram represents the proportion of respondents for each level of satisfaction on the overall QACE satisfaction scale. “Unknown” corresponding to those who did not have an opinion on the issue. On the right side, the experience of childbirth was defined as good (8 to 10), medium (5 to 7), and poor experience (0 to 4). Satisfaction is grouped according to those categories

Overall, 70.7% (*n* = 1501) of the women reported a good experience (score ≥ 8); 38% (*n* = 807) even gave their experience the maximum score of 10. On the other hand, 7.3% (*n* = 156) of women reported a poor experience, with a score < 5 (Fig. 2). Management of the delivery was very or fairly satisfactory for 94.8% (*n* = 2024). Ideal childbirth was defined as vaginal by 51.7% of the women (*n* = 1094), spontaneous labor for 37.4% (*n* = 791), and painless for 22.2% (*n* = 468). The presence of the partner improved the experience (*P* < 0.01). Delivery in a level 3 maternity ward or one with more than 3000 deliveries per year was associated with a worse experience (*P* < 0.01) (Table 2). This might be explained, on the one hand, by the description of management as unsatisfactory by 8% of the women who gave birth in level 3 hospitals (compared with 6% in level 2 and 2% in level 1 facilities) (*P* < 0.01). An alternative explanation, on the other hand, might be the type of diseases and disorders managed in the level 3 hospitals, which involve more problem or pathological pregnancies (*P* < 0.01), as well as more newborn transfers at birth (5.8% in level 3 compared with 2.2% in level 2 and 1.2% in level 1 facilities; *P* < 0.01). At the conclusion of the multivariable analysis, the variable "maternity unit level" was no longer significantly associated with the experience, because of its strong correlation with the variable "satisfaction with management" (*P* < 0.01). Reporting a pathological pregnancy appeared to be significantly associated with maternity ward level (20% in level 3 versus 12% in levels 1 and 2; *P* < 0.05).

**Table 2:** Bivariate analysis of the experience of childbirth in 3 classes, defined by the global score (score of 0 to 4, 5 to 7, and 8 to 10 out of 10)

**Variables**	**Score 0–4 (*n* = 156)**	**Score 5–7 (*n* = 464)**	**Score 8–10 (*n* = 1501)**	***P*** value
**Age < 30 years**	81 (7.9%)	224 (21.8%)	723 (70.3%)	0.69
**Age > or = 30 years**	75 (6.9%)	239 (21.9%)	774 (71.1%)	
CSP * (%):				
- Office, sales, and service staff	72 (7.1)	217 (21.2%)	732 (71.7%)	0.20
- Manager	23 (6.3%)	83 (22.7%)	259 (70%)	
- Intermediate professional	35 (9.7%)	85 (23.5%)	242 (66.9%)	
- No occupation	21 (10.6%)	45 (22.6%)	133 (66.8%)	
- Tradesperson, shopkeeper, small-business owner	3 (3.3%)	16 (20.9%)	69 (75.8%)	
- Blue-collar	2 (3.3%)	14 (23%)	45 (73.8%)	
- Farmer	0 (0%)	1 (6.7%)	14 (93.3%)	
**Maternity unit status (%):**				
- Private	32 (6.1%)	103 (19.7%)	389 (74.2%)	0.29
- Public	118 (7.8%)	347 (22.8%)	1056 (69.4%)	
- Private nonprofit	6 (9.1%)	13 (19.7%)	47 (75.1%)	
**Maternity unit level (%):**				
- 1	27 (5%)	100 (19.7%)	382 (75.1%)	** < 0.01**
- 2	84 (6.7%)	281 (22.3%)	895 (71%)	
- 3	45 (13.2%)	82 (24%)	215 (62.9%)	
**No. deliveries/year (%):**				
- < 1000	23 (5.3%)	83 (20%)	331 (75.7%)	** < 0.01**
- 1000 to 3000	70 (6.5%)	241 (22.2%)	772 (71.3%)	
- > 3000	63 (10.7%)	139 (23.6%)	389 (65.9%)	
Parity (%)				
- Nulliparous	87 (9.3%)	248 (26.5%)	601 (64.2%)	** < 0.01**
- Parous	69 (5.8%)	216 (18.2%)	900 (75%)	
**Pregnancy (%):**				
- Singleton	152 (7.4)	447 (21.7)	1468 (71%)	0.07
- Multiple	3 (10.4)	11 (37.9)	15 (51.7%)	
**Vaginal**	97 (5.3%)	351 (19.3%)	1371 (75.4%)	** < 0.01**
**Cesarean**	59 (19.7%)	112 (37.3%)	129 (43%)	
**Planned cesarean**	5 (6.6%)	21 (27.7%)	50 (65.8%)	** < 0.01**
**Cesarean was scheduled but took place at another time**	2 (8%)	8 (32%)	15 (60%)	
**Emergency cesarean**	52 (26.1%)	83 (41.7%)	64 (32.2%)	
**Particular situation**				
- Yes	28 (10%)	60 (21.4%)	193 (68.7%)	0.20
- No	127 (7%)	404 (22%)	1301 (71%)	
**Episiotomy**	20 (8.1%)	72 (29.2%)	155 (43%)	** < 0.01**
**No episiotomy**	77 (5%)	277 (17.7%)	1212 (77.4%)	
**Induction of labor**	60 (11.6%)	144 (27.9%)	313 (60.5%)	** < 0.01**
**No induction**	93 (5.9%)	317 (19.9%)	1182 (74.3%)	
**Daytime**	67 (6.9%)	215 (22%)	695 (71.1%)	0.71
**Night**	89 (7.9%)	248 (21.8%)	803 (70.4%)	
**Instrumental vaginal delivery**				
- Yes	41 (13.2%)	105 (33.9%)	164 (52.9%)	** < 0.01**
- No	56 (3.8%)	246 (16.3%)	1204 (79%)	
**Vacuum extraction**	27 (12.6%)	76 (35.4%)	112 (52.1%)	0.70
**Forceps or Spatulas**	14 (14.9%)	29 (30.9%)	51 (54.3%)	
**Epidural**	137 (22.2%)	394 (27.8%)	1152 (50%)	** < 0.01**
**No epidural**	18 (7%)	67 (21.7%)	337 (71.2%)	
**Transfer of the newborn**	12 (22.2%)	15 (27.8%)	27 (50%)	** < 0.01**
**No transfer**	144 (7%)	445 (21.7%)	1459 (71.2%)	
**Management:**^a^				
- Satisfactory	112 (5.6%)	424 (21.1%)	1475 (73.4%)	** < 0.01**
- Medium or Unsatisfactory	44 (40%)	40 (36.3%)	26 (23.7%)	
**Particular preferences expressed during the labor and delivery:**				
- Yes	57 (7.3%)	173 (22.2%)	549 (70.5%)	0.53
- No	96 (7.3%)	288 (21.9%)	930 (70.8%)	
- Don't remember	3 (12.5%)	2 (8.3)	19 (79.2%)	

*CSP Socio-professional categories

^a^satisfactory management combines "very satisfactory" and "fairly satisfactory", medium management correspond to "slightly satisfactory" and unsatisfactory management combines "fairly unsatisfactory" and "very unsatisfactory"

Vaginal delivery (aOR 3.93, 95% CI, 3.04–5.08) and satisfactory management (aOR 11.35, 95% CI, 7.69–16.75) were the principal factors determining a positive experience. The Identikit picture of the woman associated with a poor experience of her delivery was a nullipara who had a complication during her pregnancy, gave birth after induction of labor, or by cesarean or operative vaginal delivery, with the newborn transferred for pediatric care, and medical management considered unsatisfactory (Table 3). The absence of epidural analgesia improved the delivery experience (aOR 1.33, 95% CI, 1–1.78), while its presence increased the feeling of failure (aOR 5.64, 95% CI, 2.75–13.66). Pain, on the other hand, was not associated with a feeling of failure: women who graded their pain between 8 and 10 out of 10 did not have a greater feeling of failure than those who rated it between 0 and 7 of 10 (*P* = 0.13) (Table 4).

**Table 3:** Multivariable analysis explaining a positive experience of childbirth

**Positive experience of childbirth in the total population**		
**Variable**	Adjusted OR* (95% CI%)	***P*** **value**
Absence of epidural analgesia	1.33 (1–1.78)	0.05
Vaginal	3.93 (3.04–5.08)	< 0.01
No induction of labor	1.69 (1.35–2.11)	< 0.01
No neonatal transfer	1.95 (1.12–3.42)	0.02
Parous	1.48 (1.21–1.82)	< 0.01
Satisfactory management	11.35 (7.69–16.75)	< 0.01
**Positive experience of childbirth in the population with vaginal births**		
**Variable**	**adjusted OR (95% CI)**	***P*** **value**
No induction of labor	1.60 (1.23–2.06)	< 0.01
No episiotomy	1.33 (0.97–5.08)	0.07
No operative vaginal delivery	2.65 (1.98–3.55)	< 0.01
No pathological pregnancy	1.46 (1.05–2.02)	0.02
Parous	1.37 (1.07–1.82)	0.01
Satisfactory management	11.03 (7.23–16.81)	< 0.01
**Positive experience of childbirth in the cesarean population**		
**Variable**	**adjusted OR (95% CI)**	***P*** **value**
No neonatal transfer	6.49 (1.98–21.26)	< 0.01
Planned cesarean delivery took place as scheduled (vs emergency cesarean)	4.61 (1.50–9.54)	< 0.01
Planned cesarean delivery that took place at a different time (vs emergency cesarean)	3..9 (2.51–8.46)	< 0.01
No pathological pregnancy	2.61 (1.36–5.01)	< 0.01
Parous	0.58 (0.36–0.94)	0.02
Satisfactory medical management	5.46 (1.82–16.4)	< 0.01

^*^OR adjusted for variables of parity (primi- and multiparous, type of delivery, absence of epidural analgesia, no induction of labor, no neonatal transfer, satisfactory management

**Table 4:** Multivariable analysis of a feeling of failure. The feeling of failure is presented as two levels of “yes or no”

**Feeling of failure in the total population**		
**Variable**	adjusted OR* (95% CI%)	***P*** **value**
Presence of epidural analgesia	5.64 (2.75–13.66)	< 0.01
Cesarean (vs vaginal)	7.38 (5.28–10.33)	< 0.01
Induction of labor	1.82 (1.31–2.52)	< 0.01
Age as a continuous variable (+ 1 year)	0.93 (0.90–0.96)	< 0.01
Preferences expressed during pregnancy	1.61 (1.16–2.21)	< 0.01
Satisfactory management	0.16 (0.10–0.26)	< 0.01
Feeling of failure in the population with vaginal births		
**Variable**	**adjusted OR (95% CI)**	***P*** **value**
Presence of epidural analgesia	5.65 (2.22–14.40)	< 0.01
Induction of labor	1.87 (1.21–2.88)	< 0.01
No episiotomy	0.60 (0.37–1)	< 0.01
No operative vaginal delivery	0.40 (0.26–0.63)	< 0.01
Age as a continuous variable (+ 1 year)	0.95 (0.91–1)	0.05
Preferences expressed during pregnancy	1.73 (1.14–2.63)	0.01
Satisfactory management	0.14 (0.08–0.25)	< 0.01
**Feeling of failure in the cesarean population**		
**Variable**	**adjusted OR (95% CI)**	***P*** **value**
Planned cesarean delivery took place as scheduled (vs emergency cesarean)	0.18 (0.08–0.40)	< 0.01
Planned cesarean delivery that took place at a different time (vs emergency cesarean)	0.41 (0.14–1.15)	0.09
Age as a continuous variable (+ 1 year)	0.93 (0.87–0.98)	0.01

^*^OR adjusted for adjusted for variables of parity (primi- and multiparous), type of delivery, absence of epidural analgesia, no induction of labor, no neonatal transfer, satisfactory management

**Fig. 3:**
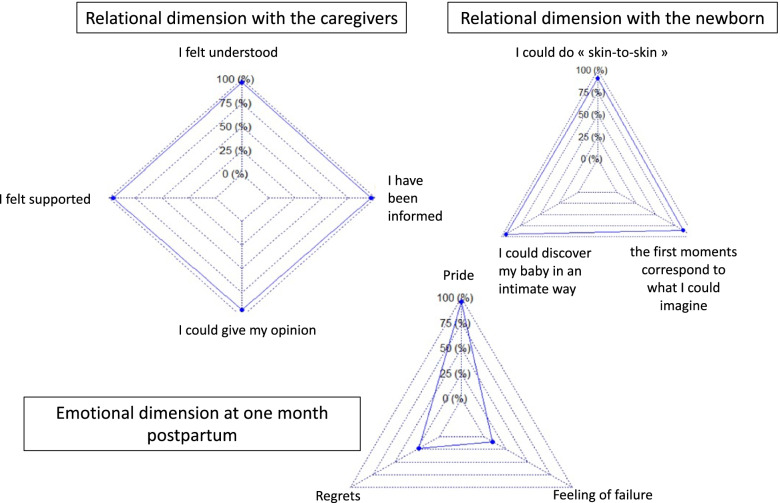
Relational dimension with caregivers and the newborn, emotional dimension at least 6 weeks postpartum

Lack of information and absence of a request for consent by health care professionals were associated with a worse experience of delivery (*P* < 0.05). There was a strong trend between the expression of particular preferences for the birth and its experience (*P* = 0.06). Information was fully satisfactory for 74.6% (*n* = 2065) of women, with 97.2% (*n* = 2075) reporting that the tone and words were appropriate. Overall 93.1% (*n* = 1982) of women were able to express and give their opinion about decisions. The health care providers understood and responded to the expectations of 96.6% (*n* = 2058) of the women (Fig. 3).

**Fig. 4:**
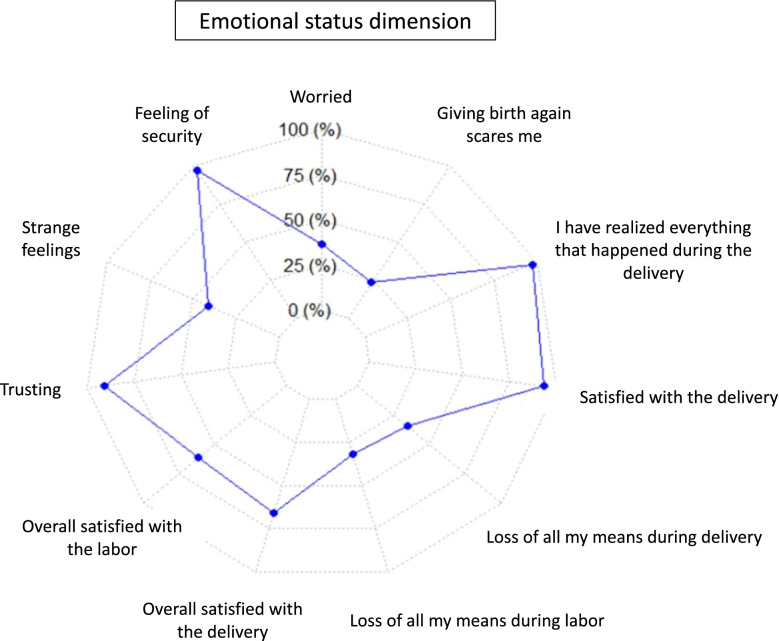
Emotional status dimension during childbirth (*n* = 2135)

Having consenting to induction of labor, for example, improved its global experience (*P* = 0.03). We also assessed the requests for consent at the moment of technical procedures: 48.1% (*n* = 149) of women with operative vaginal deliveries reported that they had not consented to its performance. A small proportion of women (6.8%, *n* = 21) reported that they had not been told that an operative intervention was about to take place, or had not been told the reason for this intervention (8.7%, *n* = 27). For episiotomies, 61.9% (*n* = 153) of the women reported that they had not consented, 46.2% (*n* = 114) that they had not been informed that it was being performed during the delivery, and 20.2% (*n* = 23) that they had not been informed even after delivery. 2.6% (*n* = 55) of the women considered that their privacy had not been respected during the delivery. Moreover, 36.6% (*n* = 780) of the women had worried during the delivery and 10.3% (*n* = 219) felt regrets (Fig. 4).

## Discussion

Our results indicate that 70% of women experienced their childbirth positively—it was a good experience. On the other hand, 7% had very negative experiences, with scores below 5/10. The literature reports positive experiences of childbirth in 66.6% to 93% of cases [5, 14–18].

We were able to identify sociodemographic data and aspects of their labor and delivery that make it possible to distinguish women who had positive experiences from those with negative experiences of giving birth. In our population, nulliparity, giving birth by cesarean, or with an operative vaginal intervention and an episiotomy, after induction of labor, and with an epidural were all associated with a worse experience, regardless of age or socio-occupational category. The quality of the relationship between the health care provider and patient was crucial in the women's experience. Our results found that the quality of management was considered best in the smallest maternity units. This finding leads us to consider recommendations related to the number of professionals required according to the size of the maternity ward, which could help to improve the quality of care. Moreover, better identification of health care staff and more information about their different roles could also contribute to improving the caregiver-patient relationship. The presence of the partner was also associated with a positive experience in our study as in the literature [17, 19].

A large retrospective Swedish cohort from 2017, including 16 775 deliveries, found a 5.7% total prevalence of women dissatisfied with their childbirth [4]. The principal risk factors for a poor experience were induction of labor, instrumental delivery, emergency cesarean delivery, severe postpartum hemorrhage, 5-min Apgar score < 7, and obstetric anal sphincter injuries. Nulliparity has also been found to be a risk factor in some studies [15, 20], although others have not found any significant association [21]. The literature about epidural analgesia is controversial, although a fair number of studies have found, as we did, that it is associated with a negative experience [17, 21]. Our study nonetheless did not differentiate between the women who had chosen to have epidural analgesia and those who had not. The mode of delivery was associated with the quality of the experience, and our results were consistent with those of other studies. Vaginal delivery was associated with a good experience [16, 22, 23], while operative vaginal deliveries and emergency cesareans were linked to a worse experience [5, 17, 22]. The literature is more mixed on the topic of planned cesarean deliveries [15–17, 20, 23]. A prolonged labor also appears to reduce satisfaction with the birthing process [3, 16, 17], as does induction of labor [24, 25].^.^ An originality of our work was our study of satisfaction according to the method of induction. We showed that initial cervical ripening reduced satisfaction, in comparison with an oxytocin infusion. On the other hand, episiotomy did not appear to be significantly associated with a poor experience in our study, after adjustment for the other risk factors (*P* = 0.07), even though it has often been identified by women to be associated with a poor experience. It has not been studied much in the literature, although one study reports a correlation with a negative experience [26].

As we have shown, the experience depends quite a lot on the care provided by the staff [14, 17, 27]. Our study did not enable us to show ethnic discrimination in the patient-caregiver relationship, as reported in one study from the USA [28] and another from Europe [29]. In the American study [28], which included 2138 women, one in six (17.3%) reported having received poor treatment during delivery, described as a loss of autonomy, a threat, or not being listened to. The negative experiences were more frequently reported to occur in hospitals, by 28.1% of women, compared with 5.1% of those who gave birth at home. The factors associated with a lower probability of poor treatment were vaginal birth, support by a midwife, and being, white, multiparous, and older than 30 years.

Our study shows clearly that providing information about procedures and asking women to consent to them before performing them improved their experience. This finding was consistent with the literature [27, 30, 31]: autonomy, that is, a sense of control during the birthing process, was a factor significantly associated with women's satisfaction [31]. This should encourage all professionals to better inform and ask women systematically for consent, even in emergency situations. In our study, 27% of the women had expressed preferences or desires during the prenatal consultations, and 9% had written a birth plan (3.7% in French national perinatal survey). There was no association between the quality of women's experiences and the preferences they expressed during pregnancy about the process of delivery (*P* = 0.53). But we found a strong trend between the birth experience and the preferences expressed as it was happening (*P* = 0.06). This was consistent with the results of a survey by a French collective of patient representatives (CIANE), in which 90% of the women whose wishes had been respected reported a good experience while giving birth. On the other hand, our study did not collect data that could have helped us to assess how preparation for childbirth affected the experience, and the literature about childbirth preparation classes is quite divergent [5, 32].

We were able to establish the dimensions of the mothers' emotional status and relationships with staff and with the newborn. The first moments with the infant [5, 15, 17, 21], and the partner's support [17, 19] were both determinant in how women experienced childbirth [33]. In the short and medium term, during the postpartum, interactions within the couple and in the mother-newborn relationship were also decisive [34].

To improve obstetric practices, WHO issued guidelines in 2016 for a positive experience of delivery, for health care focused on women and on the wellbeing of women and families [35].

This is the only French study assessing the experience of childbirth within the large population of a perinatal network. Clinical practices within the network are relatively standardized. This study is prospective, based on a validated questionnaire, with a response obtained in a predetermined amount of time after the child's birth. It thus enabled us to obtain robust data about women's experience of childbirth in France. The internal consistency of our study was validated by different associations within the questionnaire. Finally, quality of care is too often evaluated by how it meets performance objectives. This type of evaluation is not sufficient. Among the important issues involved in evaluations of the perinatal period are the need to obtain robust qualitative data, including women's satisfaction, as assessed in this study.

This study has limitations. First, it is a study based on a self-administered questionnaire. These subjective and retrospective data can include biases, related especially to emotional states and memory bias. The response period was carefully set up to limit these biases. Furthermore, half of the women who agreed to participate in the study eventually did so. This may suggest that the research group consists of women willing to share their opinions/emotions. This may give a distorted picture. Finally, we did not have access to verifiable medical data about the course of delivery: all data are self-reported. It would have been interesting to compare the staff's feelings with the experiences reported by the women.

## Conclusions

The prenatal identification of risk factors for a poor experience of delivery, improved the communication of information by professionals, and the systematic request for consent should help to improve women's experience during this essential event and thus prevent some pathological situations in the postpartum period.

## Data Availability

The datasets used and/or analysed during the current study are available from the corresponding author on reasonable request.
